# Bioinformatic Analysis of RNF43 and Its Co-expressors As Prognostic Biomarkers for Gastric Cancer

**DOI:** 10.7759/cureus.98422

**Published:** 2025-12-03

**Authors:** Masanobu Tsubaki, Taira Matsuo, Rie Komori, Haruka Kubo, Chihiro Miyamoto, Noriaki Nagai

**Affiliations:** 1 Laboratory of Pharmacotherapy, Tokushima Bunri University, Takamatsu, JPN; 2 Department of Pharmacy, Kindai University, Osaka, JPN

**Keywords:** axin2, bioinformatics analysis, biomarker, gastric cancer, hunk, overall survival, rnf43

## Abstract

Background

Gastric cancer (GC) exhibits high morbidity and mortality rates worldwide. Although surgery, pharmacotherapy using antibody drugs, and chemotherapy improve the survival rate, some patients exhibit a poor prognosis, necessitating the identification of new prognostic factors. Downregulation or mutation of the ring finger protein (RNF)-43, a negative regulator of the Wnt pathway, serves as a poor prognostic factor for various cancers; however, its specific mechanisms in GC remain unclear. Therefore, in this study, we used a clinical database to determine whether RNF43 and its co-expressors impact the life expectancy of patients with GC.

​Methods

The evaluations were executed on publicly available datasets and website for analysis, including the University of Alabama at Birmingham Cancer Data Analysis Portal (UALCAN), cBioPortal, Kaplan-Meier plotter, Linkedomics, and WebGestalt.

Results

Notably, high RNF43 expression prolonged the overall survival (OS) of patients with GC. AXIN2, zinc and ring finger three (ZNRF3), bone morphogenetic protein and activin membrane-bound inhibitor (BAMBI), and hormonally upregulated Neu-associated kinase (HUNK) were identified as RNF43 co-expressors. Among these, co-expression of ZNRF3 and BAMBI had no impact, whereas co-expression of AXIN2 prolonged the patient's OS. In contrast, overexpression of HUNK alone was associated with a poor prognosis for patients with GC. Moreover, overexpression of both RNF43 and HUNK was associated with prolonged OS compared to the overexpression or unaltered expression of HUNK alone.

Conclusion

Collectively, these results suggest RNF43 and AXIN2 as favorable prognostic factors and HUNK as a poor prognostic factor and therapeutic target for patients with GC.

## Introduction

Gastric cancer (GC) ranks fifth in cancer incidence and fourth in cancer-related mortality worldwide [1]. Gastrectomy with D2 lymphadenectomy and perioperative or adjuvant therapy is the global standard of care for locally progressive GC [2,3]. Despite the gradual improvement in survival rate after radical therapy, over half of the patients with localized progressive GC exhibit recurrence and limited survival rates post-recurrence [4]. Currently, chemotherapy with platinum-based drugs, such as oxaliplatin and cisplatin, and fluoropyrimidines, such as 5-fluorouracil (5-FU), capecitabine, and S-1, is the standard treatment for advanced GC [5,6]. Additionally, drugs targeting GC-related factors (claudin 18.2, human epidermal growth factor receptor 2 (HER2), and programmed cell death-1), such as zolbetuximab (anti-claudin 18.2 monoclonal antibody), trastuzumab (anti-HER2 monoclonal antibody), and pembrolizumab (anti-programmed cell death-1 monoclonal antibody), are used in combination with chemotherapy. These drugs improve the response rates of patients with advanced GC harboring the above-mentioned targets [7-9]. Despite the advances in therapy, the prognosis of patients with GC remains poor [10], underscoring the need for new prognostic biomarkers and therapeutic targets for GC.

Ring finger protein (RNF)-43 is an E3 ubiquitin ligase that negatively regulates the Wnt/β-catenin signaling pathway [11,12]. Some RNF43 mutations cause loss of function, promote β-catenin stabilization, and induce tumorigenesis; RNF43 mutations are frequently observed in gastric, ovarian, pancreatic, endometrial, colon, and liver cancers [13,14]. Mutations in RNF43 have been identified in about 10% of patients with GC [14]. In addition, the diminished expression and loss-of-function mutations of RNF43 in GC cells enhance cell invasion and proliferation, while also conferring resistance to 5-FU, cisplatin, and radiation-induced DNA damage by mitigating their effects [15]. Furthermore, RNF43 mutations in GC have been shown to be frequently observed in tumors exhibiting microsatellite instability [16]. Moreover, it has been observed that RNF43 expression in GC cells negatively correlates with PD-L1 expression and that high RNF43 expression enhances T cell antitumor activity [17]. These findings suggest that reduced expression or loss-of-function mutations in RNF43 might be implicated in resistance to chemotherapy or immune checkpoint inhibitors in GC and may serve as useful prognostic predictors.

In this study, we analyzed whether RNF43 is a potential prognostic factor for GC and whether its co-expressed genes influence the prognosis, utilizing publicly available databases.

## Materials and methods

UALCAN analysis

University of Alabama at Birmingham Cancer Data Analysis Portal (UALCAN) database, a platform for cancer data analytics utilizing information from The Cancer Genome Atlas (TCGA), was used to examine the differential expression of RNF43 mRNA in gastric tumor tissues [18]. The analysis of this database was conducted under the following parameters: "TCGA Gene: RNF43", "dataset: stomach adenocarcinoma", "Links for analysis: Expression", and "Sample types: individual cancer stages". The analysis conducted under these circumstances produced the following outcomes: stage 1 (N=18), stage 2 (N=123), stage 3 (N=169), and stage 4 (N=41).

cBioPortal

Changes in RNF43 mRNA expression levels in patients with GC were analyzed using the cBioPortal [19]. RNF43 mRNA overexpression (RNA Seq V2 RSEM) was defined by a Z-score of ± 2.0. This analysis utilized the "TCGA, PanCancer Atlas" dataset. By applying the aforementioned Z-score to differentiate between RNF43 mRNA high and unaltered categories, the results were high (N=23) and unaltered (N=384). Kaplan-Meier curves were used to analyze the associations between RNF43 gene expression changes and the overall survival (OS), progression-free survival (PFS), and disease-specific survival (DSS) of patients with GC.

Kaplan-Meier plotter

The Kaplan-Meier plotter was used to determine the predictive value of the RNF43 gene for patients with GC [20]. To analyze overall survival (OS) and relapse-free survival (RFS) based on RNF43 mRNA expression, a dataset from "Pan-cancer RNA-seq" for "Stomach adenocarcinoma" was utilized. GC patients were classified into high- and low-expression groups based on RNF43 mRNA expression levels using data calculated via Kaplan-Meier plots (auto select best cutoff set to percentiles). Kaplan-Meier curves for OS and RFS were generated using this data. In the OS analysis, GC patients were divided into RNF43 high (N=95) and RNF43 low (N=276) groups. For the RFS analysis, the groups consisted of high (N=130) and low (N=85). Regarding the effect of HUNK mRNA expression in the OS, analysis was performed using "Affy id: 1555935_s_at and 219535_at" based on "mRNA in gastric cancer". For 1555935_s_at, HUNK expression was high (N=360) and low (N=271); for 219535_at, it was high (N=80) and low (N=551).

Co-expression analysis

For RNF43 mRNA co-expression analysis, we identified and investigated the top 1000 genes exhibiting positive co-expression with RNF43 mRNA using cBioPortal and Linkedomics [21]. In cBioPortal, after confirming RNF43 mRNA expression using the "Stomach Adenocarcinoma (TCGA, PanCancer Atlas)" dataset, we selected the "Co-expression" tab and chose the top 1000 mRNAs co-expressed with RNF43. In Linkedomics, after selecting "RNA-Seq" and 'RNF43' using the "Stomach Adenocarcinoma" dataset in the Cancer Cohort, we selected "RNA-Seq" in the target dataset and "Pearson Correlation test" in the statistical method to list the top 1000 mRNAs co-expressed with RNF43. To further understand the features and pathways related to RNF43, Gene Ontology (GO) and Kyoto Encyclopedia of Genes and Genomes (KEGG) enrichment analyses were conducted using WebGestalt [22]. Enrichment analysis was performed for RNF43 co-expressed mRNAs identified in both cBioPortal and Linkedomics. Additionally, mRNAs positively correlated with RNF43 mRNA expression (r≥0.4 and Benjamini-Hochberg corrected p-value <0.0001) were identified using cBioPortal.

Statistical analyses

Statistical significance was analyzed via two-sided t-test and analysis of variance, followed by Dunnett's test. Kaplan-Meier curves and log-rank analyses were used to evaluate the patient survival rates. The significance level was set at 5%.

## Results

Association of RNF43 expression with the clinical stage of patients with GC

**Figure 1:**
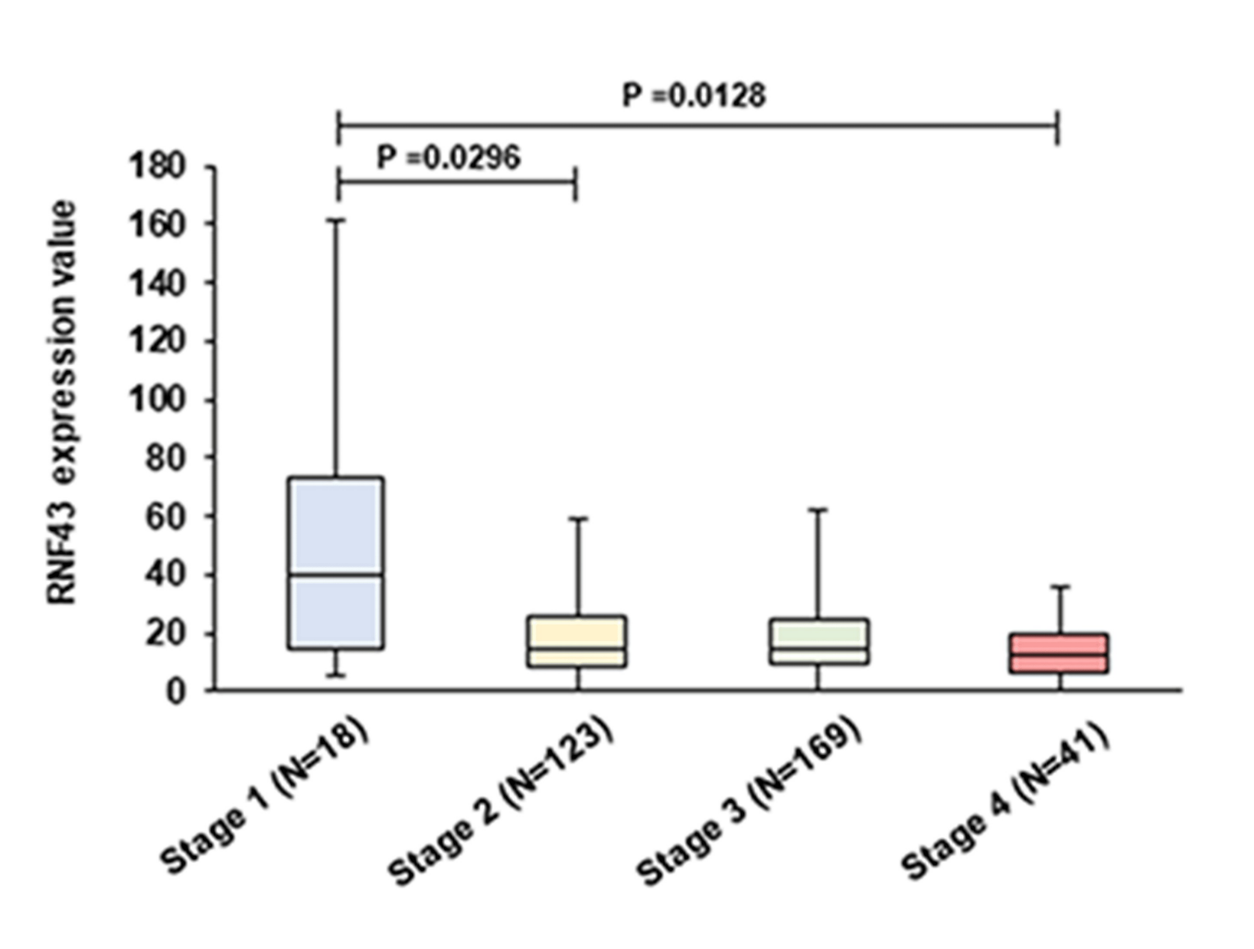
Comparison of RNF43 expression levels in patients with gastric cancer by clinical stage using the UALCAN database UALCAN - University of Alabama at Birmingham Cancer Data Analysis Portal; RNF - ring finger protein

We investigated whether RNF43 expression correlated with the tumor stage of patients with GC using the UALCAN database. RNF43 expression decreased as the tumor stage progressed (Figure 1).

High RNF43 mRNA expression is correlated with the prognosis of patients with GC

**Figure 2:**
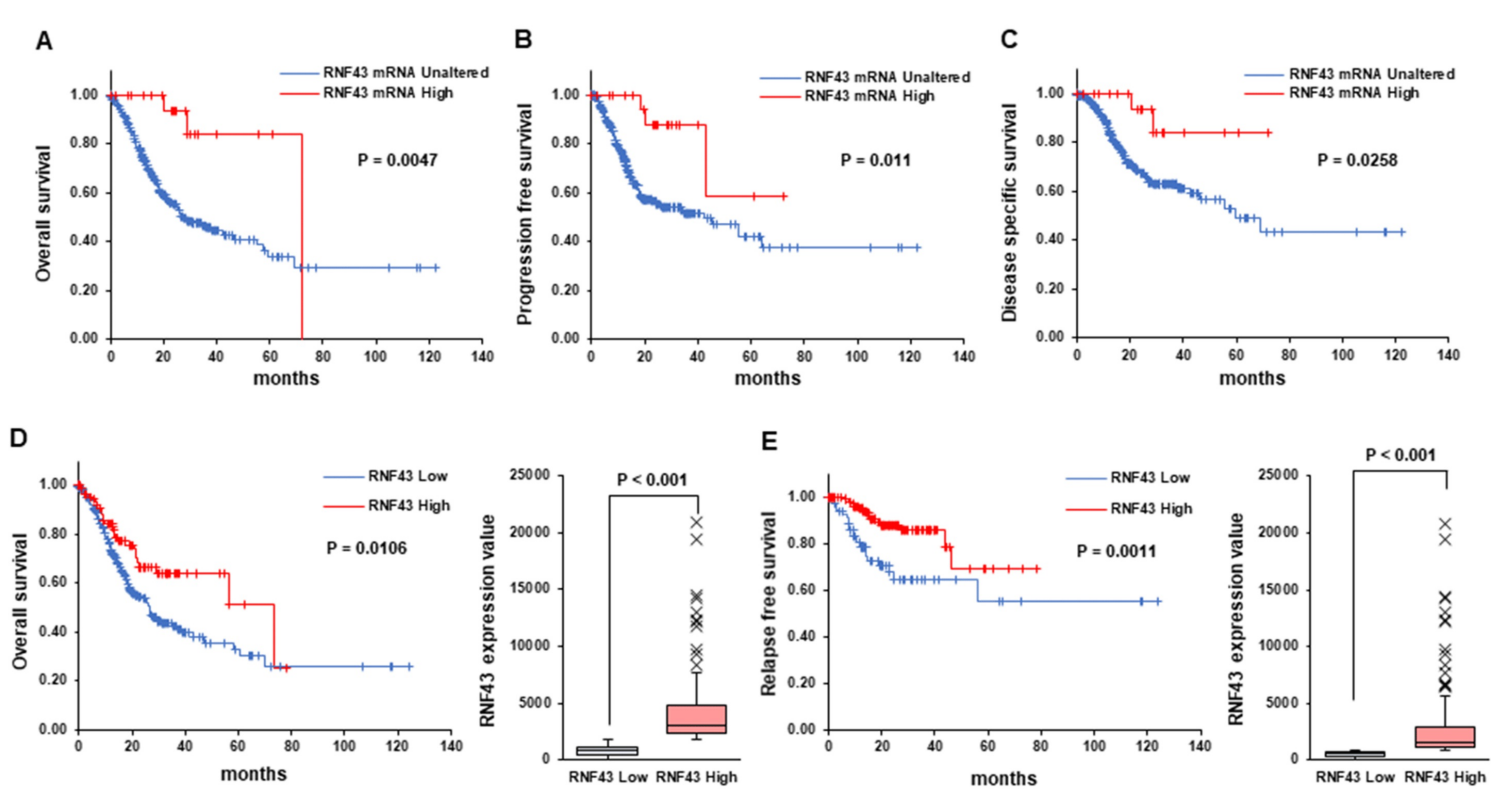
Prolonged life expectancy of patients with gastric cancer with high RNF43 expression levels (A-C) Overall (A), progression-free (B), and disease-specific (C) survival of patients with gastric cancer with high RNF43 levels were analyzed using the cBioPortal. (D and E) Correlations of RNF43 expression with the overall and relapse-free survival rates of patients with gastric cancer were analyzed using the Kaplan-Meier plotter. (D) Correlation between RNF43 expression and overall survival of patients with gastric cancer. (E) Correlation between RNF43 expression and relapse-free survival of patients with gastric cancer. RNF - ring finger protein

Next, the prognostic significance of RNF43 mRNA expression in patients with GC was evaluated using the cBioPortal and Kaplan-Meier plotter. cBioPortal analysis revealed that patients with high RNF43 mRNA levels exhibited potentially better OS, PFS, and DSS than those with low RNF43 mRNA levels (Figure 2A-C). Kaplan-Meier plotter analysis also showed that patients with high RNF43 mRNA levels may exhibit a better prognosis, including higher OS and relapse-free survival (RFS), than those with low RNF43 mRNA levels (Figure 2D and E). These findings suggest high RNF43 mRNA levels may be useful as a prognostic biomarker for patients with GC.

Analysis of RNF43 mRNA co-expressed genes and their functional enrichment in GC

**Figure 3:**
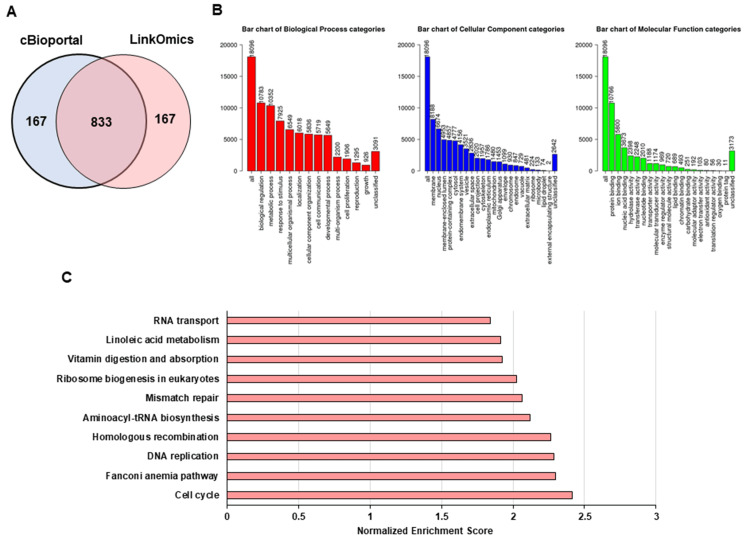
Comprehensive bioinformatics analysis of RNF43 and its co-expressed genes in gastric cancer (A) Intersection of positively co-expressed genes among the top 1000 genes identified using cBioPortal and LinkedOmics. (B and C) Gene Ontology (GO) and Kyoto Encyclopedia of Genes and Genomes (KEGG) enrichment analyses of RNF43 RNF - ring finger protein

Top 1000 genes positively co-expressed with RNF43 were screened using cBioPortal and LinkedOmics (Figure 3A). GO and KEGG enrichment analyses were conducted to predict the RNF43 functions and pathways. Biological process terms included growth, cell proliferation, cell communication, and metabolic processes. Cellular component terms included the cytosol, nucleus, and membrane. Molecular function terms involved transferase activity, hydrolase activity, ion binding, and protein binding (Figure 3B). KEGG pathways included the cell cycle, Fanconi anemia, DNA replication, and mismatch repair pathways (Figure 3C). These results suggest that the RNF43 mRNA co-expressed genes may be involved in various cancer suppression-related processes. Therefore, RNF43 may play an important role in suppressing GC progression.

**Figure 4:**
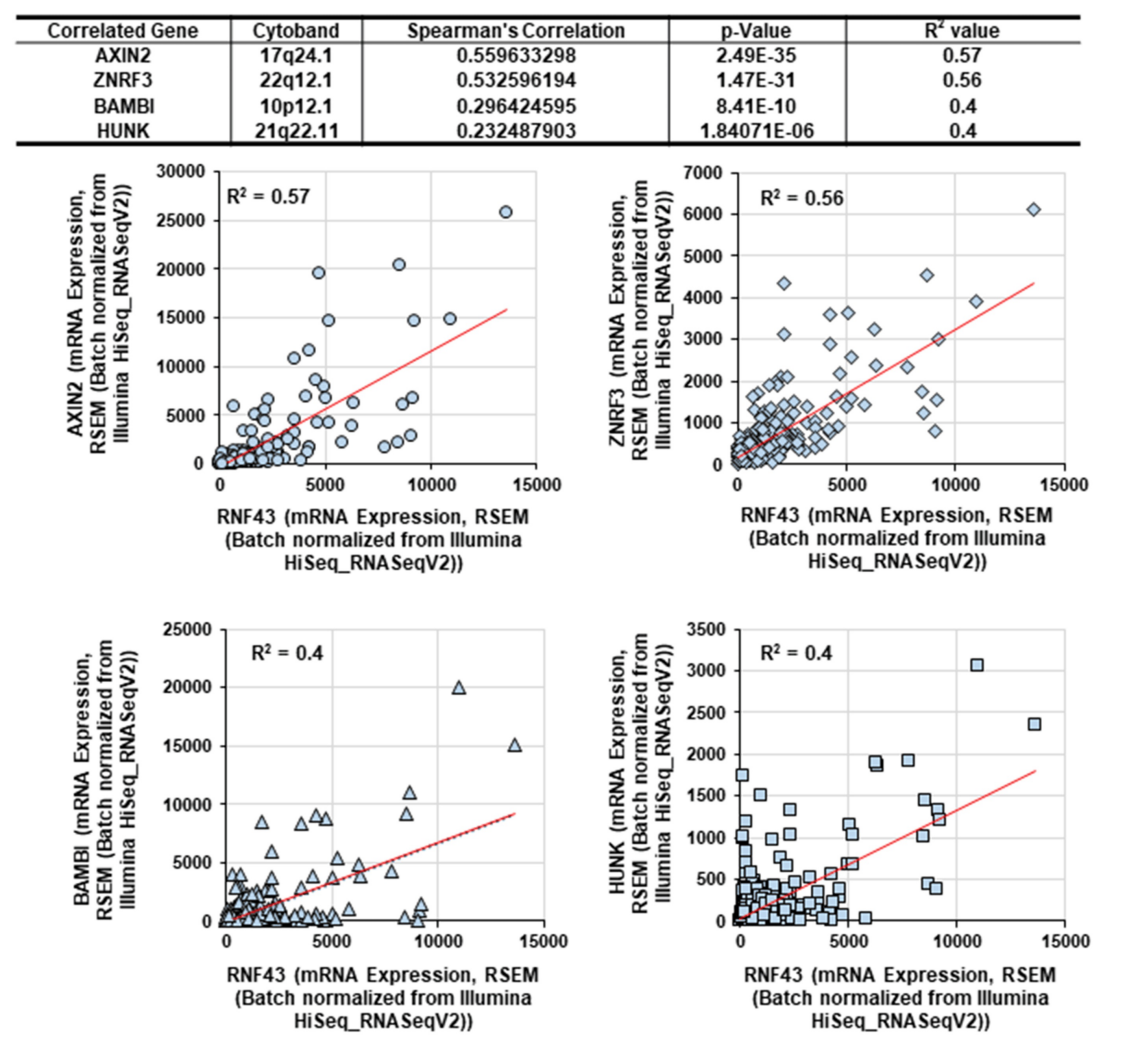
RNF43 co-expressor analysis of patients with gastric cancer using the cBioPortal Four factors showing significant differences with R2 values >0.4 were identified.

Next, we determined the factors correlated with RNF43 mRNA expression among the co-expressed genes identified using the cBioPortal. Among the identified genes (Figure 3A), AXIN2, zinc and ring finger 3 (ZNRF3), bone morphogenetic protein and activin membrane-bound inhibitor (BAMBI), and hormonally upregulated neu-associated kinase (HUNK) mRNA levels exhibited R2 values ≥0.4 (Figure 4). This suggests that RNF43 may suppress tumor progression in cooperation with the above-mentioned genes.

Predictive value of the factors co-expressed with RNF43

**Figure 5:**
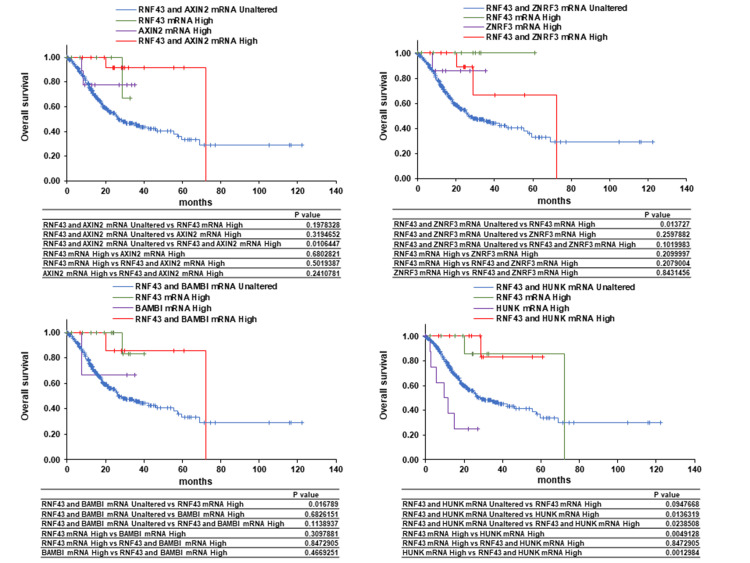
Effect of RNF43 co-expression with AXIN2, ZNRF3, BAMBI, or HUNK on the overall survival of patients with gastric cancer RNF - zinc and ring finger three; ZNRF3 - zinc and ring finger three; BAMBI - bone morphogenetic protein and activin membrane-bound inhibitor; HUNK - hormonally upregulated Neu-associated kinase

**Figure 6:**
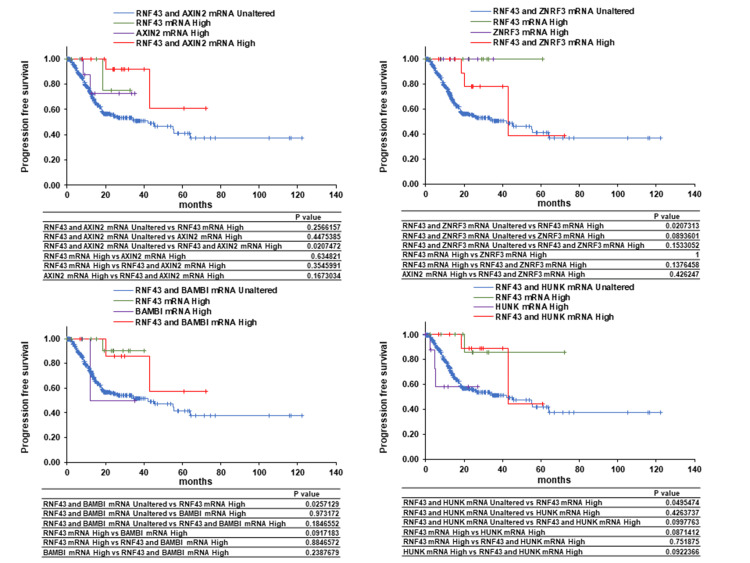
Effect of RNF43 co-expression with AXIN2, ZNRF3, BAMBI, or HUNK on the progression-free survival of patients with gastric cancer RNF - zinc and ring finger three; ZNRF3 - zinc and ring finger three; BAMBI - bone morphogenetic protein and activin membrane-bound inhibitor; HUNK - hormonally upregulated Neu-associated kinase

**Figure 7:**
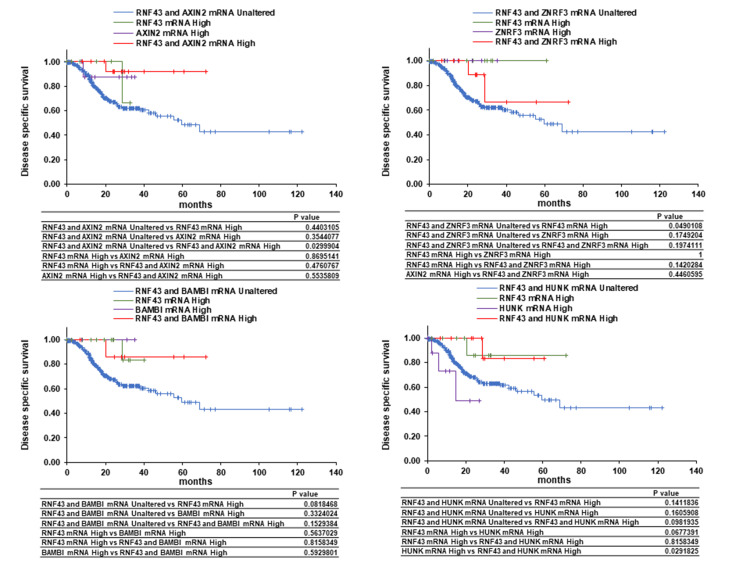
Effect of RNF43 co-expression with AXIN2, ZNRF3, BAMBI, or HUNK on the disease-specific survival of patients with gastric cancer RNF - zinc and ring finger three; ZNRF3 - zinc and ring finger three; BAMBI - bone morphogenetic protein and activin membrane-bound inhibitor; HUNK - hormonally upregulated Neu-associated kinase

**Figure 8:**
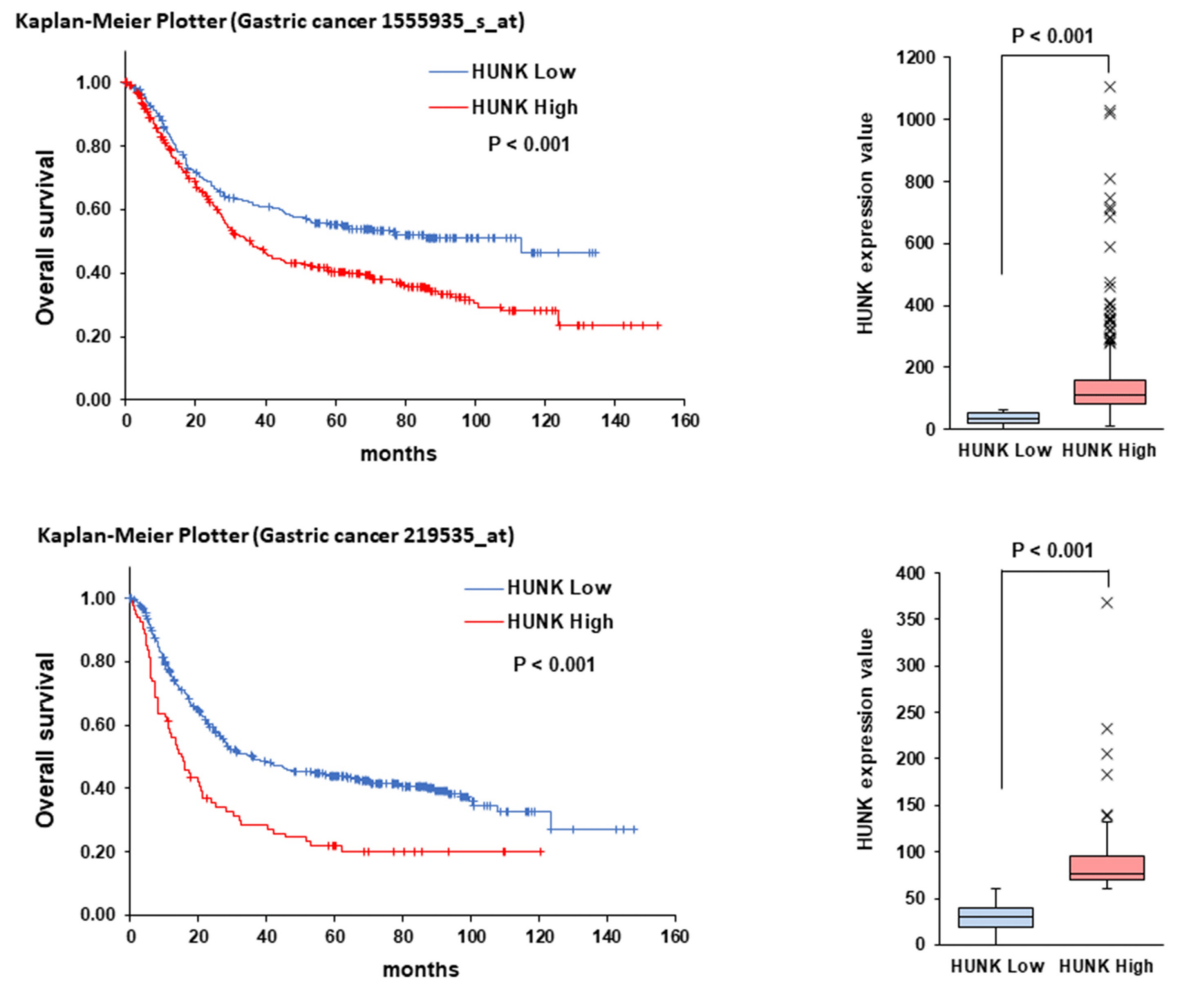
Correlation of HUNK expression with the overall survival of patients with gastric cancer determined using the Kaplan-Meier plotter (ID: 1555935_s_at and 219535_at) HUNK - hormonally upregulated Neu-associated kinase

Correlations of the mRNA levels of RNF43 and its co-expressors, AXIN2, ZNRF3, BAMBI, and HUNK, with the OS, PFS, and DSS of patients with GC were analyzed using the cBioPortal. Overexpression of both RNF43 and AXIN2 was found to be potentially associated with prolonged OS. No significant differences in OS were observed between RNF43 and ZNRF3 co-expression and between RNF43 and BAMBI co-expression. High expression of HUNK was found to potentially shorten OS, whereas RNF43 and HUNK co-expression and high RNF43 expression were confirmed to potentially prolong OS (Figure 5). Similar trends were observed for PFS and DSS (Figure 6, Figure 7). These findings suggest that AXIN2 may function as a favorable prognostic factor in GC patients in concert with RNF43, while HUNK may act as an unfavorable prognostic factor (Figure 8). Therefore, RNF43 likely suppresses HUNK, which is probably an unfavorable prognostic factor.

## Discussion

In this study, RNF43 mRNA expression decreased with GC progression, and OS, PFS, DSS, and RFS were possibly shorter in patients with low or unchanged RNF43 mRNA levels than in those with high RNF43 mRNA levels. RNF43 expression is associated with the tumor-node-metastasis stage, distant metastasis, and survival rate of patients with GC, with decreased RNF43 expression supporting the cancer stem cell-like maintenance of GC cell lines [23]. RNF43 overexpression abrogates the Wnt5A-induced drug resistance and tumor progression and improves the OS in patients with melanoma [24]. Notably, RNF43 expression is related to OS, possibly serving as a prognostic predictor for patients with glioma [25]. These findings suggest that high RNF43 expression may prolong the OS, PFS, DSS, and RFS of patients with GC.

RNF43 expression is associated with the prognosis of various cancers and enhances the sensitivity to immunotherapy, conventional anticancer drugs, and molecular-targeted drugs by affecting signal transduction, drug metabolism, and the tumor immune microenvironment [26]. In this study, RNF43 was involved in various cell growth regulatory mechanisms and processes, such as cell proliferation, metabolic processes, transferase activity, and mismatch repair. Additionally, AXIN2, ZNRF3, BAMBI, and HUNK were identified as RNF43-expressors. AXIN2, ZNRF3, and BAMBI are target genes of β-catenin, with AXIN2 negatively regulating the Wnt/β-catenin pathway by degrading β-catenin [27,28]. ZNRF3 is another negative regulator of Wnt/β-catenin; similar to RNF43, it degrades Frizzled, a Wnt receptor [29]. BAMBI acts as an inhibitor of the transforming growth factor-β type 1 receptors, playing a promoting role in advanced tumors and an inhibitory role during early tumor development [30]. HUNK is implicated in tumorigenesis, metastasis, and invasion via epidermal growth factor receptor activation in HER2-positive and triple-negative breast cancers [31,32]. In this study, overexpression of both RNF43 and AXIN2 demonstrated the potential to improve the OS, PFS, and DSS of patients with GC compared to the overexpression or unaltered expression of RNF43 and AXIN2 alone. Homeobox C6 overexpression reduces the RNF43 and AXIN2 expression levels, leading to a poor prognosis for patients with colorectal cancer [33]. Furthermore, low RNF43 and AXIN2 levels indicate the involvement of β-catenin activation in inflammatory bowel disease-associated colorectal cancer development [34]. Borealin overexpression enhances tumor growth and metastasis by downregulating the RNF43 and AXIN2 expression levels and activating β-catenin, thereby decreasing the survival rate of patients with hepatocarcinoma [35]. Compared to high AXIN2 levels, low AXIN2 levels are associated with the shorter survival of patients with lung and pancreatic cancers [36,37]. These findings suggest that low RNF43 and AXIN2 levels may be poor prognostic factors and potential prognostic predictors for patients with GC.

Overexpression of HUNK alone was possibly associated with shorter OS compared to the overexpression of both RNF43 and HUNK or no change in expression. A similar decreasing trend was observed in PFS and DSS. Compared to low HUNK expression, high HUNK expression may be associated with a poor prognosis in patients with GC. HUNK expression is also associated with the distant metastasis-free survival and OS of patients with triple-negative breast cancer [31]. Additionally, HUNK is associated with HER2-targeted therapy resistance of HER2-positive breast cancer cells [38]. These findings suggest that HUNK may be a potential therapeutic target for GC.

This study has several limitations. First, this study relied on a clinical database and did not directly assess the data of patients with GC. Second, in vitro and in vivo studies using GC cell lines were not performed to validate the obtained results. Third, the specific relationship between RNF43 and HUNK expression levels could not be determined. Fourth, since this study utilizes public databases, detailed data on GC patients are not available. Consequently, multivariate analysis, assessment of dataset heterogeneity, and subgroup analysis based on multiple mRNA expressions could not be performed. Therefore, further studies are necessary to address these limitations and validate our findings.

## Conclusions

In summary, RNF43 overexpression was shown to potentially prolong the OS, PFS, DSS, and RFS of patients with GC. AXIN2 and HUNK were identified as potential co-expression factors with RNF43. Specifically, RNF43 co-expression with AXIN2 was found to potentially improve the patients' OS. In contrast, overexpression of HUNK alone was shown to potentially shorten the patients' OS. Moreover, overexpression of both RNF43 and HUNK may reduce the effect of HUNK and potentially enhance the patients' OS compared to no change in expression. Overall, our clinical database analysis indicated that RNF43 and AXIN2 overexpression may be a favorable prognostic factor, whereas HUNK overexpression may be a poor prognostic factor, suggesting potential therapeutic targets for patients with GC.

## References

[1] Sung H, Ferlay J, Siegel RL, Laversanne M, Soerjomataram I, Jemal A, Bray F (2021). Global Cancer Statistics 2020: GLOBOCAN Estimates of Incidence and Mortality Worldwide for 36 Cancers in 185 Countries. CA Cancer J Clin.

[2] Lordick F, Carneiro F, Cascinu S (2022). Gastric cancer: ESMO Clinical Practice Guideline for diagnosis, treatment and follow-up. Ann Oncol.

[3] Ajani JA, D'Amico TA, Bentrem DJ (2022). Gastric Cancer, Version 2.2022, NCCN Clinical Practice Guidelines in Oncology. J Natl Compr Canc Netw.

[4] Janjigian YY, Ajani JA, Moehler M (2024). First-line nivolumab plus chemotherapy for advanced gastric, gastroesophageal junction, and esophageal adenocarcinoma: 3-year follow-up of the Phase III Checkmate 649 trial. J Clin Oncol.

[5] Kang YK, Chin K, Chung HC (2020). S-1 plus leucovorin and oxaliplatin versus S-1 plus cisplatin as first-line therapy in patients with advanced gastric cancer (SOLAR): a randomised, open-label, phase 3 trial. Lancet Oncol.

[6] Al-Batran SE, Homann N, Pauligk C (2019). FLOT4-AIO Investigators. Perioperative chemotherapy with fluorouracil plus leucovorin, oxaliplatin, and docetaxel versus fluorouracil or capecitabine plus cisplatin and epirubicin for locally advanced, resectable gastric or gastro-oesophageal junction adenocarcinoma (FLOT4): a randomised, phase 2/3 trial. Lancet.

[7] Shitara K, Lordick F, Bang YJ (2023). Zolbetuximab plus mFOLFOX6 in patients with CLDN18.2-positive, HER2-negative, untreated, locally advanced unresectable or metastatic gastric or gastro-oesophageal junction adenocarcinoma (SPOTLIGHT): a multicentre, randomised, double-blind, phase 3 trial. Lancet.

[8] Kawakami T, Yamazaki K (2024). Recent progress in treatment for HER2-positive advanced gastric cancer. Cancers (Basel).

[9] Mansoor H, Gohar M, Attaria A, Karim FF, Naeem U, Khan M, Iqbal J (2025). Evaluating comparative effectiveness of pembrolizumab-based therapy versus chemotherapy in treatment of gastric carcinoma: a systematic review and meta-analysis of randomized controlled trials. Clin Exp Med.

[10] Li K, Zhang A, Li X, Zhang H, Zhao L (2021). Advances in clinical immunotherapy for gastric cancer. Biochim Biophys Acta Rev Cancer.

[11] Nusse R, Clevers H (2017). Wnt/β-catenin signaling, disease, and emerging therapeutic modalities. Cell.

[12] Koo BK, Spit M, Jordens I (2012). Tumour suppressor RNF43 is a stem-cell E3 ligase that induces endocytosis of Wnt receptors. Nature.

[13] Nag JK, Appasamy P, Malka H, Sedley S, Bar-Shavit R (2024). New target(s) for RNF43 regulation: implications for therapeutic strategies. Int J Mol Sci.

[14] Wang K, Yuen ST, Xu J (2014). Whole-genome sequencing and comprehensive molecular profiling identify new driver mutations in gastric cancer. Nat Genet.

[15] Neumeyer V, Brutau-Abia A, Allgäuer M (2021). Loss of RNF43 function contributes to gastric carcinogenesis by impairing DNA damage response. Cell Mol Gastroenterol Hepatol.

[16] (2014). Comprehensive molecular characterization of gastric adenocarcinoma. Nature.

[17] Yang J, Wei M, Liu X (2023). PD-L1 expression downregulation by RNF43 in gastric carcinoma enhances antitumour activity of T cells. Scand J Immunol.

[18] Chandrashekar DS, Karthikeyan SK, Korla PK (2022). UALCAN: An update to the integrated cancer data analysis platform. Neoplasia.

[19] de Bruijn I, Kundra R, Mastrogiacomo B (2023). SC Analysis and visualization of longitudinal genomic and clinical data from the AACR Project GENIE Biopharma Collaborative in cBioPortal. Cancer Res.

[20] Győrffy B (2024). Integrated analysis of public datasets for the discovery and validation of survival-associated genes in solid tumors. Innovation (Camb).

[21] Vasaikar SV, Straub P, Wang J, Zhang B (2018). LinkedOmics: analyzing multi-omics data within and across 32 cancer types. Nucleic Acids Res.

[22] Liao Y, Wang J, Jaehnig EJ, Shi Z, Zhang B (2019). WebGestalt 2019: gene set analysis toolkit with revamped UIs and APIs. Nucleic Acids Res.

[23] Gao Y, Cai A, Xi H (2017). Ring finger protein 43 associates with gastric cancer progression and attenuates the stemness of gastric cancer stem-like cells via the Wnt-β/catenin signaling pathway. Stem Cell Res Ther.

[24] Radaszkiewicz T, Nosková M, Gömöryová K (2021). RNF43 inhibits WNT5A-driven signaling and suppresses melanoma invasion and resistance to the targeted therapy. Elife.

[25] Xi S, Zhang X, Chen H (2015). Downregulation of ring-finger protein 43 in glioma associates with poor prognosis. Int J Clin Exp Pathol.

[26] Xu Y, Lin Z, Ji Y, Zhang C, Tang X, Li C, Liu T (2023). Pan-cancer analysis identifies RNF43 as a prognostic, therapeutic and immunological biomarker. Eur J Med Res.

[27] Katoh M (2018). Multi‑layered prevention and treatment of chronic inflammation, organ fibrosis and cancer associated with canonical WNT/β‑catenin signaling activation (Review). Int J Mol Med.

[28] Li S, Wang C, Liu X, Hua S, Liu X (2015). The roles of AXIN2 in tumorigenesis and epigenetic regulation. Fam Cancer.

[29] Hao HX, Jiang X, Cong F (2016). Control of Wnt receptor turnover by R-spondin-ZNRF3/RNF43 signaling module and its dysregulation in cancer. Cancers (Basel).

[30] Weber F, Treeck O, Mester P, Buechler C (2023). Expression and function of BMP and activin membrane-bound inhibitor (Bambi) in chronic liver diseases and hepatocellular carcinoma. Int J Mol Sci.

[31] Williams CB, Phelps-Polirer K, Dingle IP (2020). HUNK phosphorylates EGFR to regulate breast cancer metastasis. Oncogene.

[32] Dilday T, Abt M, Ramos-Solís N (2024). Identification and characterization of a potent and selective HUNK inhibitor for treatment of HER2+ breast cancer. Cell Chem Biol.

[33] Qi L, Chen J, Zhou B (2021). HomeoboxC6 promotes metastasis by orchestrating the DKK1/Wnt/β-catenin axis in right-sided colon cancer. Cell Death Dis.

[34] Rajamäki K, Taira A, Katainen R (2021). Genetic and epigenetic characteristics of inflammatory bowel disease-associated colorectal cancer. Gastroenterology.

[35] Chen B, Gu Y, Shen H (2022). Borealin promotes tumor growth and metastasis by activating the Wnt/β-catenin signaling pathway in hepatocellular carcinoma. J Hepatocell Carcinoma.

[36] Dai F, Zhu LJ, Zhang W (2019). The association between three AXIN2 variants and cancer risk. J Cell Biochem.

[37] Cao W, Dai S, Ruan W, Long T, Zeng Z, Lei S (2023). Pancreatic stellate cell-derived exosomal tRF-19-PNR8YPJZ promotes proliferation and mobility of pancreatic cancer through AXIN2. J Cell Mol Med.

[38] Phelps-Polirer K, Abt MA, Smith D, Yeh ES (2016). Co-targeting of JNK and HUNK in resistant HER2-positive breast cancer. PLoS One.

